# Evaluation of selected thrombotic factors among pregnant women with preeclampsia and normal pregnant women

**Published:** 2014-12

**Authors:** Nafiseh Saghafi, Atieh Mohammadzadeh Vatanchi, Fatemeh Tara, Leila Pourali, Salmeh Dadgar

**Affiliations:** 1 *Department of Obstetrics and Gynecology, Qaem Hospital, Mashhad University of Medical Sciences, Mashhad, Iran.*; 2 *Department of Obstetrics and Gynecology, Mashhad University of Medical Sciences, Mashhad, Iran. *; 3 *Department of Obstetrics and Gynecology, Om-albanin Hospital, Mashhad University of Medical Sciences, Mashhad, Iran. *

**Keywords:** *Preeclampsia*, *Thrombotic factors*, *Pregnancy*

## Abstract

**Background:**

Preeclampsia is one of the common complications during pregnancy with considerable maternal and fetal mortality and morbidity. Hypercoagulability due to thrombophilic factors is discussed as the etiology involved in this disease.

**Objective:**

The aim of this study was to evaluate selected thrombotic factors among pregnant women with preeclampsia and normal pregnant women.

**Materials and Methods:**

This case-control study was performed on 200 pregnant women at third trimester of pregnancy between 2012 and 2013. 100 pregnant women admitted to Qaem and Imam Reza hospitals of Mashhad, due to preeclampsia, were selected as case group and 100 pregnant women without preeclampsia referred to OB/GYN clinic of these hospitals as control group. Blood samples were taken from two groups for evaluation of the coagulation factors including factor V Leiden, protein C, protein S, antithrombin III, anti-cardiolipin antibodies, and lupus anticoagulant antibodies.

**Results:**

Two groups were not significantly different in terms of maternal age and parity (p>0.05). Levels of factor V Leiden, protein C, protein S, antithrombin III, anti-cardiolipin antibodies and lupus anticoagulant antibodies were compared between two groups. The number of patients with abnormal factor V Leiden and protein C was significantly higher in case group than in the control group (p<0.01 respectively), but other factors were not significant different between two groups. Thrombophilia disorders were significantly more in case group compared to control (p<0.001).

**Conclusion:**

The risk of thrombophilia disorders is higher in preeclamptic patients than normal pregnant women.

## Introduction

Preeclampsia as the most common medical complication during pregnancy (5-8%) affects 2-7% of all pregnancies and is a major cause of maternal and fetal mortality that is defined as the incidence of hypertension ≥140/90 after 20 weeks of pregnancy along with proteinuria (- 4). However, underlying etiologic mechanisms remain poorly understood (5). During the last decade, the identification of several gene polymorphisms that are associated with hypercoagulability (inherited thrombophilia) has advanced understanding of etiologic mechanisms of thrombosis (6, 7). Also, proteomics analyses of pregnant women with preeclampsia have enlightened the fact that the expression of different proteins including those that play a role in hemostasis and coagulation cascades, as well as metabolic processes, are significantly altered in preeclampsia (8).

In a two-step hypothesis of preeclampsia, vascular disorders have been suggested as the initiator factor and a series of factors as catalyst that thrombophilic disorders are included. In recent years, some studies have suggested that inherited thrombophilia, may be associated with preeclampsia (-). Therefore, the effect of thrombophilic factors on the severity or causing preeclampsia, eclampsia and complications during pregnancy has been discussed in various studies. Based on the study type and studied factors and geographical areas, controversial results have been obtained (13). Proposed underlying mechanisms include interference with trophoblast differentiation, inadequate placentation, or thrombosis of placental vasculature, with consequent reduced placental perfusion, oxidative stress, and maternal endothelial dysfunction that is believed to trigger the hallmark biological and clinical manifestations of preeclampsia (14, 15).

$n=\frac{[p_{1}\left(1-p_{1}\right)+p_{2}(1-p_{2})]{\left(Z_{1-\frac{\alpha }{2}}+Z_{1-\beta }\right)}^{2}}{{\left(p_{1}-p_{2}\right)}^{2}}=\frac{(0.167\times 0.833+0.037\times 963){\left(1.96+0.84\right)}^{2}}{{\left(0.167-0.37\right)}^{2}}=81$

Important thrombophilic factors in preeclampsia that have been studied so far include factor V Leiden, protein C, protein S, antithrombin III, anti-cardiolipin antibodies, and lupus anticoagulant antibodies (16). A review study reported that the women with preeclampsia have more thrombophilic disorder than the women in control group (17). Also, in a study conducted in 2004, it was showed that at least one thrombophilia factor disorder was seen in 40-72% of patients with preeclampsia compared with 8-20% in the control group (18). However, a large prospective observational study conducted in 2005 found no association between the mutation factor V Leiden and preeclampsia (19). Since the race and ethnicity play important role in the prevalence of thrombophilic factors, and so far, no studies are performed on the prevalence of these factors and their relation to preeclampsia in this area, therefore, we decided to conduct a study in this field.

Thus, the aim of this study was to evaluate selected thrombotic factors among pregnant women with preeclampsia and normal pregnant women. Based on these results, we could offer the required suggestions about the need for screening of coagulation factors in this geographical area and also, the efficacy of anticoagulant in the treatment of preeclampsia. 

## Materials and methods

This case-control study was performed from the beginning of October 2012 to October 2013, on 200 pregnant women: A hundred pregnant women who due to preeclampsia were admitted in Qaem and Imam Reza hospitals of Mashhad, were selected as case group and a hundred pregnant women in their third trimester who referred to obstetrics and gynecology clinics of aforementioned hospitals for their routine checkup were designated as control group. Patients were considered to have preeclampsia if they had blood pressure ≥140/90 mm Hg and developed proteinuria (≥0.3 gr in a 24 hour urine collection) after the 20^th^ week of gestation (4). 

Our sample size was estimated based on the study performed by Giorgio Mello *et al *(16). In their study, 3.7% of controls and 16.7% of cases were either +/+ or +/- for Factor V Leiden. According to this results and by considering 95% confidence interval and α=0.05, sample size was calculated:

In order to increase the confidence, we used a hundred patients for case group and a control was enrolled for each (200 patients in total). mAll patients were informed and written consents were taken. Patients who did not want to participate and all patients in control group with a history of abortion, preterm labor, thrombophlebitis, pulmonary embolism or preeclampsia were excluded from the study. We used a questionnaire to obtain the demographic data and blood samples were taken to evaluate the following factors:

1. Lupus anticoagulant antibodies
2. Factor V Leiden, which was evaluated by measurement of Activated Protein C Resistance ratio, with considering values >2 as normal and values <1.8 as abnormal.
3. Protein C activity (normal range: 70-160%)
4. Protein S total activity, with a normal range of 55-160 %.
5. Anti-cardiolipin antibodies. IgM and IgG antibodies were measured and values >15 U/ml were considered as abnormal.
6. Antithrombin III, with a normal range of 80-125 %.

In order to evaluate protein C, S, and factor V Leiden, STEELEX was used and antithrombin III was measured by ELISA and anti-cardiolipin by Chrous.

**Statistical analysis**

In order to analyze our data we employed the analytics software, SPSS® version 11.5 and the chi-square test was used. P<0.05 were considered to be statistically significant.

## Results

The mean age of total patients in the study was 24.94 with a standard deviation of 3.59 years that two groups showed no significant difference (p=0.48). Also, evaluation of the patients’ parity showed no significant difference between two groups (p=0.82) (Table I). The values of the factors measured in this study are shown in Table II. Measurements of the factor V Leiden showed that the level of this factor in the control group was 2.51±0.38 and in the case group was 2.72±0.61. Abnormal values were observed in 12 patients (12%) in case group. Chi-square test showed that two groups were significantly different in terms of abnormal values of factor V Leiden (p<0.01). 

The activated protein C resistance ratio was considered to be abnormal for values <1.8, which observed in twelve cases (12%). Abnormal values of protein S levels were four patients (4%) in control group and 6 (6%) in case group that the difference was not statistically significant (p=0.36). The measurement of antithrombin III showed abnormal values in six patients (6%) of control group and 12 (12%) of case group and no significant difference was found between two groups (p=0.14). 

The number of abnormal values of anti-cardiolipin antibodies was four patients (4%) in control group and three (3%) in case group that there was no significant difference between two groups in terms of abnormal values of anti-cardiolipin antibodies (p=0.70). There was no positive case of Lupus anticoagulant antibodies in both groups, therefore, the difference was not statisticaly significant between two groups (p=1.00).

**Table I:** Comparison of the age and parity in total patients and in two groups

		**Case group**	**Control group**	**Total**	**p-value** *
**Age (years)** *		24.87 ± 3.47	25.3 ± 3.71	24.94 ± 3.59	0.489
**Parity** **					
	One	73 (73%)	72 (72%)	145 (72.5%)	0.823
	Two	14 (14%)	17 (17%)	31 (15.5%)	
	Three	8 (8%)	6 (6%)	14 (7%)	
	Four	1 (1%)	2 (2%)	3 (1.5%)	

*: data are presented as mean±SD.

**: data are presented as n (%)

**Table II:** The levels of factors in total patients and in two groups

**Factors**	**Case group**	**Control group**	p-value*
anti-cardiolipin antibody	2.66 ± 3.46	2.82 ± 2.18	0.7
antithrombin III	101.9 ± 15.04	103.49 ± 18.01	0.143
Protein S	94.95 ± 36.80	83.03 ± 25.24	0.363
Protein C	88.36 ± 32.70	100.12 ± 20.62	0.002
Factor V Leiden	2.72 ± 0.61	2.51 ± 0.38	0.009
lupus anticoagulant antibody	0	0	1

Data are presented as mean±SD.

## Discussion

Pathologic study of preeclampsia-eclampsia has suggested that hypercoagulability is considered as catalyst, therefore, the effect of thrombophilic factors on the severity or causing preeclampsia, eclampsia and adverse events during pregnancy has been considered in different studies (11, 20, 21). In our study there was significant difference in terms of the amount of factor V Leiden and protein C and showed that the possibility of lack of factor V Leiden and protein C was more in women with preeclampsia than other pregnant women without preeclampsia. It was also shown that thrombophilia markers disorder was higher in preeclampsia group.

Also, Alfirevic and colleagues evaluated the women with pregnancy complications (including eclampsia/ preeclampsia, fetal death, congenital incidence of thrombophilia) and showed that congenital thrombophilia was more observed in the group with adverse outcomes. Abnormal thrombophilic factors were observed in 54 patients of case group and 17 patients in control group. The number of abnormal thrombophilic factors was more in their study compared to our study so that screening of abnormal thrombophilic was positive in 31% of our cases, but in the study of Alfirevic *et al* was 53% (22). 

In a review study performed by Alfirevic *et al*, deficiency of protein C, S, and factor V Leiden was higher in preeclampsia group than normal pregnancy group that their results are similar to our results (17). In another inquiry performed by Karimi *et al* 8.6% of cases (pregnant women with preeclampsia) and 1% of controls (healthy women) showed the factor V Leiden mutation and it was concluded that pregnant women with this mutation are prone for preeclampsia (23). In that respect, the results of aforementioned study is similar to our results.

In a large case-control study in 2005 by Mello and colleagues, 808 white patients with preeclampsia were compared with 808 patients who had passed pregnancy without any incidence and no difference was observed between two groups in terms of age and parity. Women with severe preeclampsia (406 cases) were at higher risk for hereditary or acquired thrombophilic factors except for protein C, S and antithrombin deficiency (16). However, in our study, decreased protein C was more observed in preeclampsia group than control group. In addition, acute renal failure, Disseminated Intravascular Coagulation (DIC), abortion and fetal death in these patients was higher than in patients without coagulation abnormalities. An important advantage of this study compared to our study is the higher sample size and also, attention to the severity of preeclampsia in statistical analysis and the group with severe preeclampsia has been individually evaluated and actually, no significant difference was observed in mild preeclampsia group.

Similar to the study of Mello, the study of Facchinetti and colleagues at Italy in June 2009 evaluated the coagulation factors (factor V Leiden, protein S, protein C, deficiency of antithrombin III, anticardiolipin antibodies, lupus anti-coagulant antibodies, hyperhomocysteinemia, factor II G20210A and MTHFR C677) in 172 pregnant women with a history of preeclampsia; 60 patients (34%) had thrombophilic deficiency and the risk of preeclampsia and preterm delivery (under 32 weeks) was higher in these patients than the mothers without deficiency (ratio=2.5); their findings were similar to our study (24). In a recent inquiry performed by Ogawa *et al* plasma antithrombin levels were evaluated in women with gestational hypertension and preeclampsia (25). 

In above-mentioned study, authors have concluded that antithrombin III activity in gestational hypertension and preeclampsia is correlated with the serum albumin and total protein, which suggests that antithrombin III activity decreases in this condition. Consequently the preeclampsic women are more prone to thrombosis. After numerous studies performed to confirm the significant relation between thrombophilic factors and preeclampsia, other two large studies showed different results (-). A multicenter cohort study in Montreal was conducted to examine whether thrombophilia increases the risk of preeclampsia. They reported that thrombophilia was shown in 14% of patients with preeclampsia and 21% of control group and concluded that there was no evidence that supports the relation between hereditary thrombophilia and preeclampsia (29).

In a study by Livingstone and colleagues, the prevalence of hereditary genotypes mutation associated to thrombophilia with severe preeclampsia has been evaluated. In this prospective study, the prevalence of factor V Leiden mutation, prothrombin, and MTHFR (Methylenetetrahydrofolate reductase) was evaluated. 110 patients with severe preeclampsia were compared with 97 patients with similar gestational age who had no problem in terms of preeclampsia. Evidence showed no significant differences between patients with severe preeclampsia and control groups in terms of factor V Leiden (G/506/A mutation), MTHFR (CC/667/TT mutation) and prothrombin (G/20210/A). Also, there was no association between severe pre-eclampsia and fetal thrombophilia; they concluded that there is no relationship between severe preeclampsia and inherited thrombophilia (30). 

Since this analysis was conducted on genotype of complicated patients, it is considered a unique study. These results show that despite of our study and similar studies above-mentioned, there are other studies that are controversial for the effect of thrombophilia disorders on the incidence of preeclampsia. Therefore, this recommendation that the tests for diagnosing thrombophilia would have been a marker for preeclampsia is approved.

**Limitations**

According to many previous studies, it seems that thrombophilic disorders lead to preterm (early onset) and severe preeclampsia that in our study, the severity and time of causing preeclampsia are not considered. Since the follow-up of this study has been performed until delivery, in the case of complication to preeclampsia in the first few days after delivery is not included in the results. Scientifically, thrombophilic disorders should be confirmed by re-sampling that it was not possible for us due to financial limitations.

## Conclusion

Preeclampsia is from the common medical complication of pregnancy that one of its accelerating factors is hypercoagulability through thrombophilic factors. Consistent with the findings of our study and other studies, it was shown that the probability of thrombophilia disorders is higher in patients with preeclampsia, but further studies are needed to prove it as a prognostic factor.

**Recommendation**

Since we concluded that thrombophilic disorders are considerable in our region and can lead to pregnancy vascular complications, it is recommended to perform a study with more extensive context about the prevalence of these disorder and association with pregnancy complications in order to be a background for interventional clinical studies about that whether using of anticoagulants can be effective in prophylaxis of pregnancy complications in thrombophilic patients or not. It is recommended that in further studies, the severity and time of onset of the disease, other pregnancy vascular complications and neonatal outcomes be also considered and the effect of anticoagulants in the treatment of thrombophilic patients be evaluated.
